# Duration of the pubertal growth spurt in patients with increased craniofacial growth component in sagittal and vertical planes—retrospective and cross-sectional study

**DOI:** 10.1007/s00784-021-03799-7

**Published:** 2021-02-05

**Authors:** Agnieszka Szemraj-Folmer, Anna Wojtaszek-Słomińska, Bogna Racka-Pilszak, Małgorzata Kuc-Michalska

**Affiliations:** 1 Department of Orthodontics, Faculty of Medicine, Medical University of Gdańsk, Al.Zwyciestwa 42c, 80-210 Gdańsk, Poland; 2 Private Orthodontic and Dental Clinic, ul.Pawliczka 10/1, 41-800 Zabrze, Poland

**Keywords:** Growth spurt, Growth spurt duration, Cervical vertebral maturation, Cephalometric measurements

## Abstract

**Objectives:**

The aim of the study is to assess the skeletal age at the onset and end of the pubertal growth spurt and determine its duration in four growth type groups: (1) normodivergent skeletal Class I (I N), (2) normodivergent skeletal Class III (III N), (3) high-angle skeletal Class III (III H) and (4) high-angle skeletal Class I (I H).

**Materials and methods:**

Two hundred thirteen subjects were selected from 2163 examined files. The cervical vertebral maturation stage was recorded by means of Baccetti’s method. The sagittal and vertical skeletal relations were evaluated according to Steiner analysis with Kaminek’s modification. The duration of the pubertal growth spurt was calculated from the difference between the means of the chronological age related to CS3 and CS4 maturation stages.

**Results:**

The shortest lasting pubertal growth spurt was observed in group I N (1.1), followed by group III N (1.6). Major differences between arithmetic means CS4-CS3 were seen in groups I H and III H (2.3 and 2.7, respectively).

**Conclusions:**

The following tendency was observed in the duration of the pubertal growth spurt: I N < III N < I H < III H. This tendency has statistical significance only in high-angle patients in comparison with normodivergent skeletal Class I.

**Clinical relevance:**

Knowledge on the longer pubertal growth spurt in high-angle patients compared to patients with normal anteroposterior and vertical relationships can be useful in the selection of an appropriate therapeutic method and a treatment time.

**Supplementary Information:**

The online version contains supplementary material available at 10.1007/s00784-021-03799-7.

## Introduction

In humans, pubertal growth spurt is a period of many, often intense, changes in the body. Observation of a juvenile patient in this period can significantly impact the choice of a suitable orthodontic-orthopaedic therapeutic method. If treatment is deferred at the time, the growth potential may be irreversibly lost [1]. For this reason, assessment of the skeletal age when patients with orthodontic problems are diagnosed is so important [2, 3].

**Fig. 1:**
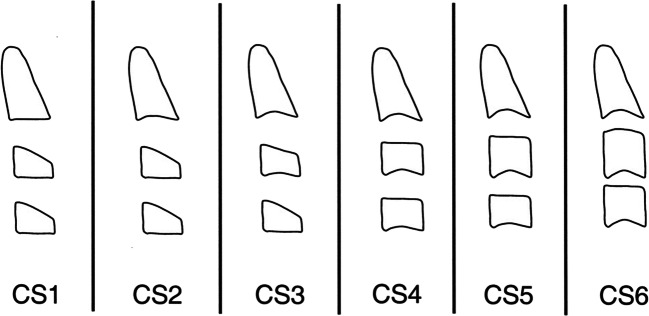
Cervical vertebral maturation method according to Baccetti, Franchi and McNamara (2005). Source: Baccetti T, Franchi L, McNamara JA Jr. (2005) The cervical vertebral maturation (CVM) method for the assessment of optimal treatment timing in dentofacial orthopedics. Seminars in orthodontics 11:119–129

A number of methods can be used to determine the skeletal age. To evaluate the patient’s maturation stage, one can examine a radiograph of the hand and the wrist [4], the knee [5] or a cephalometric x-ray picture. Methods heretofore used to analyse the morphology of the vertebrae have been modified many times and improved [6, 7]. The most recent modification of the cervical vertebral maturation (CVM) stages is the one proposed in 2005 by Baccetti, Franchi and McNamara (Fig. 1) [8]. It is this classification which has gained many supporters and opponents. The main objection is that CVM method is characterised by lack of repeatability and lack of specific features that can be used to define each stage [9, 10]. There are studies, however, that confirm both repeatability and correlation between CVM and HWM [11–13]. The CVM method does not require additional radiographic documentation since the vertebrae are examined on a cephalometric radiograph—a standard for diagnosing malocclusions [8, 14]. The hand-wrist method, accepted as the “gold standard”, involves taking a radiograph, whose sole purpose is to determine the bone age. It has to be stressed that during the therapeutic process of the growing patient, a determination of the skeletal age—which often does not correlate with the chronological age [15–17]—needs to be performed several times. The benefit of the CVM method is the elimination of unnecessary exposure of the patient to radiation—a vital element in today’s radiological protection. According to the authors, the most intense mandibular growth, which occurs during the pubertal growth spurt, occurs between the CS3 and CS4 stage [8].

The pubertal growth spurt is characterized by dynamic mandibular growth and less noticeable maxillary growth [8, 18]. Significant gender differences can be observed in this period; therefore, all possible deviations have to be considered individually [1]. Females mature faster, by about 2 years, than males [19]. Generally speaking, maturation stages in boys are more difficult to pinpoint than in girls because they are less specific [20]. Other factors also significantly impact the maturation process, namely hormonal changes, the environment, genetics, climate and nutrition [21]. Studies indicate that the pubertal growth spurt is also different in populations with various types of skeletal malocclusion. The most frequently observed correlation can be defined as follows: the pubertal growth spurt is shorter in skeletal Class II and takes longer in Class III in comparison with patients with normal anteroposterior and vertical cephalometric measurements [22–24]. However, studies on pubertal growth spurt in patients with vertical plane disorders are still scarce [25]. Due to vertical craniofacial growth, which lasts longer than the transverse or the sagittal ones, it was important to check whether the increased vertical growth component is reflected in the duration of the pubertal growth spurt [26].

The aim of the study is to assess the skeletal age at the onset and end of the pubertal growth spurt and determine its duration in four groups: normodivergent skeletal Class I; normodivergent skeletal Class III; high-angle, skeletal Class III (with skeletal open bite); and high-angle skeletal Class I (with skeletal open bite).

## Material and methods

This is a retrospective, cross-sectional study.

Ethical approval was obtained from The Independent Bioethics Committee at The Medical University in Gdańsk prior to data collection (NKBBN/43/2017). This study was performed in line with the principles of the Declaration of Helsinki.

The sample size was calculated by the G*Power software, version 3.1.9.7. Basing on the pilot study, the following assumptions were made: a two-tailed test with a power of 90%, a significance level of *p* = 0.05, the effect size d of 1 and an allocation ratio of 1. The minimal sample size was 23 individuals in one group. A total of 213 patients qualified for the study; the least numerous group comprised 25 individuals.

A database of cephalometric radiographs (2163 in total) was reviewed in order to collect the study material. The radiographs were taken of patients who presented at the Department of Orthodontics, Medical University of Gdańsk and Private Orthodontic and Dental Clinic in Zabrze, Poland between 2008 and 2019. When making the selection, the most important common criterion was CS3 stage (which correlated with the onset of the pubertal growth spurt) or CS4 (indicating the end of the pubertal growth spurt) acc. to the CVM six maturation stages proposed by Baccetti et al. [8]. Patients with concavity at the base of C2 and C3 were qualified for stage CS3, provided the concavity at C4 was absent. C3 and C4 were trapezoid in shape or resembled a horizontally positioned rectangle. For the CS4 stage, patients manifesting C2, C3 and C4 concavity were qualified. C3 and C4 vertebrae had the shape of a horizontally positioned rectangle. Other inclusion criteria included age (7–18 years) and good quality radiographs. Patients who had previously undergone orthodontic treatment, had clefts or genetic disorders were excluded from the study. Skeletal criteria acc. to Steiner’s analysis with Kaminek’s modification defining individual groups were: normodivergent skeletal Class I–WITS = 0 ± 2 mm, NS/ML = 33 ± 6° (I N); normodivergent skeletal Class III–WITS < − 2 mm, NS/ML = 33 ± 6° (III N); high-angle skeletal Class III–WITS < − 2, NS/ML > 39° (III H); high-angle Class I–WITS = 0 ± 2 mm, NS/ML > 39° (I H).

The first selection of radiographs produced 768 pictures. Of this total, a number of radiographs were excluded due to 151—other skeletal discrepancies (i.e. Class II or deep bite), 189—inadequate CVM stage, 123—incomplete orthodontic records, 92—genetic disorders or clefts. Eventually, 213 radiographs were obtained, which were allocated to eight groups including the following number of patients: I N, CS3–29 patients; I N, CS4–29; III N, CS3–25; III N, CS4–25; III H, CS3–28; III H, CS 4–25; I H, CS3–25; I H, CS4–27. No single patient was categorized as both CS3 and CS4 stages.

**Fig. 2:**
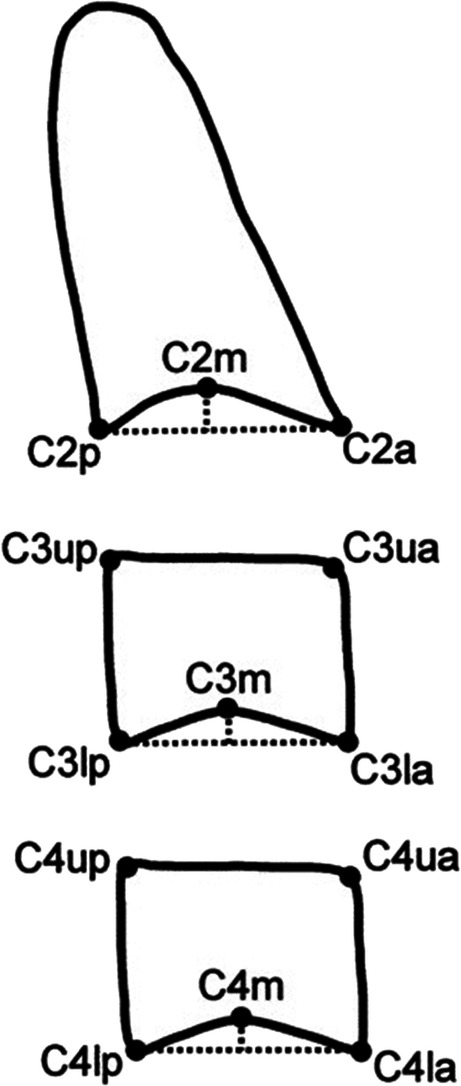
Vertebral points, measures and ratios used to determine the vertebral stage in the CVM method. C3BAR: ratio between the length of the base (distance C3lp-C3la) and the anterior height (distance C3ua-C3la) of the body of C3. C3PAR: ratio between the posterior (distance C3up-C3lp) and anterior (distance C3ua-C3la) heights of the body of C3. C4BAR: ratio between the length of the base (distance C4lp-C4la) and the anterior height (distance C4ua-C4la) of the body of C4. C4PAR: ratio between the posterior (distance C4up-C4lp) and anterior (distance C4ua-C4la) heights of the body of C4. Source: Baccetti T, Franchi L, McNamara JA Jr. (2005) The cervical vertebral maturation (CVM) method for the assessment of optimal treatment timing in dentofacial orthopedics. Seminars in orthodontics 11:119–129

The following parameters were adopted when cephalometric radiographs were being taken with Gendex Ortoralix 9200: 68–72 kV, 8 mA and 1 s. In order to eliminate any measurement error, the CVM stages and cephalometric measurements were assessed by two independent researchers at 1-month interval. The two obtained parameters served to calculate the arithmetic mean. Assessment of three cervical vertebrae was made with Baccetti’s method, which takes into consideration the depth of C2m, C3m and C4m concavity (the concavity had to represent at least 10% of the posterior height of the vertebral structure), as well as the value of C3BAR, C3PAR, C4BAR and C4PAR parameters (Fig. 2) [8]. The intra- and interobserver agreement was expressed by kappa coefficient. The values of kappa gave a result of 0.92 (inter-) and 0.94 (intra-) observer agreement.

In cases when there were discrepancies in classification of CVM stages, the researchers reached agreement by means of exchange of opinions and discussion.

### Statistical analysis

All statistical calculations were made by means of the R software, version 3.6.3. (2020) and Excel 2010 spreadsheet. Quantitative variables were characterized by means of the arithmetic mean and standard deviation. To check whether the examined variable for each group comes from a normal distribution, the Shapiro-Francia test for normality (with Benjamini-Hochberg’s modification) was used. Group homogeneity was confirmed with Levene’s analysis. The significance of the differences between stage CS3 and CS4 for each type of malocclusion was calculated using the Student’s *t* test for independent samples. Arithmetic mean in different groups for stages CS3 and CS4 were compared using the ANOVA and post-hoc Tukey tests. The duration of the pubertal growth spurt was calculated from the difference between the means of the chronological age related to CS3 and CS4. Next, the duration of the pubertal growth spurt was compared between groups by means of a linear model with interaction effects. For all the tests, the significance level was set at *p* < 0.05.

## Results

A total of 213 cephalometric radiographs were accepted for the study including 129 female and 84 male patients. The evaluation of the bone age at the onset and end of pubertal growth spurt has been presented here using descriptive statistics (Table 1). The lowest mean for the CS3 stage was recorded for groups III H and I N (10.72. and 10.74, respectively), and for the CS4 stage also in group I N (11.81). For patients in group III N, the growth spurt started the last (11.94), and ended the last in I H (13.76). The maximum age in stage CS4 was the highest in high-angle patients (I H and III H–17.42). Separate calculations for both genders have also been included in tables; they are supposed to add a perspective since the requirement of the minimal number of patients in a given group was not met. The most significant gender differences were observed in group III H—the onset of pubertal growth spurt occurred even 2 years later in males than in females, and ended 1 year later at the most.

**Table 1:** Descriptive statistics

Gender	Type of malocclusion	CVM stage	No. of records	Mean age [year]	Standard deviation	Minimum age [year]	Maximum age [year]
Female + Male	I N	CS3	29	10.74	0.94	9	12
		CS4	29	11.81	1.41	9.33	14.92
	III N	CS3	25	11.94	1.47	9.92	14.75
		CS4	25	13.5	1.42	11	16.33
	III H	CS3	28	10.72	1.7	7.42	13.58
		CS4	25	13.4	1.81	10.08	17.42
	I H	CS3	25	11.49	1.51	8.75	14.5
		CS4	27	13.76	1.86	9.5	17.42
Female	I N	CS3	19	10.57	1.01	9	11.75
		CS4	17	11.5	1.24	9.33	14.5
	III N	CS3	13	11.17	1.51	9.92	14
		CS4	13	13.19	1.16	11.92	14.92
	III H	CS3	20	10.16	1.56	7.42	12.67
		CS4	14	12.95	0.93	11.5	14.5
	I H	CS3	14	11.14	1.4	8.75	13.67
		CS4	19	13.82	1.85	9.5	17.25
Male	I N	CS3	10	11.07	0.71	10.08	12
		CS4	12	12.26	1.57	10.75	14.92
	III N	CS3	12	12.54	1.2	11.25	14.75
		CS4	12	13.85	1.64	11	16.33
	III H	CS3	8	12.11	1.19	9.92	13.58
		CS4	11	13.96	2.48	10.08	17.42
	I H	CS3	11	11.95	1.58	9.42	14.5
		CS4	8	13.63	2.03	11.75	17.42

In all the groups, arithmetic mean for stage CS3 significantly differed statistically from those for stage CS4 (Table 2).

**Table 2:** The comparison of mean ages between CS3 and CS4 stages in groups with different types of malocclusion

		I N	III N	III H	I H
Male + Female	CS3	10.74	11.94	10.72	11.49
	CS4	11.81	13.5	13.4	13.76
	*p* value	0.001*	< 0.001*	< 0.001*	< 0.001*
Female	CS3	10.57	13.38	10.16	11.14
	CS4	11.58	13.19	12.92	13.82
	*p* value	0.019*	0.002*	< 0.001*	< 0.001*
Male	CS3	11.07	12.54	12.11	11.95
	CS4	12.26	13.85	13.96	13.63
	*p* value	0.031*	0.037*	0.069*	0.058*

In order to compare stage CS3 and CS4 between all the groups, the ANOVA analysis of variance was performed. Whenever the result was statistically significant, the post-hoc (Tukey HSD test for normal distribution) was used to check the differences between particular groups (Table 3). The mean chronological age for stage CS3 did not show significant statistical differences between groups defined according to types of malocclusion in males and females. However, when all the data were compared, statistically significant differences were observed between groups I N and III N as well as III N and III H. The arithmetic means for stage CS4 differed significantly for all participants and for females in all groups when malocclusions were compared with the group I N. In males, no statistically significant differences were noted for stage CS4.

**Table 3:** The comparison of mean ages at CS3 and CS4 stages among groups with different types of malocclusion. ANOVA

Gender	CVM stage	Mean age				*p* value	Post-hoc Tukey HSD					
		I N	III N	III H	I H		I N vs. III N	I N vs. III H	I N vs. I H	III N vs. III H	III N vs. I H	III H vs. I H
Male + Female	CS3	10.74	11.94	10.72	11.49	0.004*	0.014*	1	0.219	0.013*	0.689	0.204
	CS4	11.81	13.5	13.4	13.76	< 0.001*	0.001*	0.003*	< 0.001*	0.996	0.941	0.853
Female	CS3	10.57	11.38	10.16	11.14	0.061						
	CS4	11.5	13.19	12.95	13.82	< 0.001*	0.008*	0.025*	< 0.001*	0.972	0.586	0.295
Male	CS3	11.07	12.54	12.11	11.95	0.057						
	CS4	12.26	13.85	13.96	13.63	0.141						

The shortest lasting pubertal growth spurt was observed in group I N (1.07), followed by group III N (1.56) in which it lasted 6 months longer. Major differences between arithmetic means CS4-CS3 were seen in groups I H and III H (2.27 and 2.68, respectively). Both groups of high-angle patients (I H and III H) differed significantly from the normodivergent skeletal Class I (I N). In other comparative assessments, no statistically significant differences were observed (Table 4).

**Table 4:** The comparison of mean duration (in years) of pubertal growth spurt for different types of malocclusion. A linear model with interaction variables. CS4-CS3 – the difference between the means of the chronological age related to CS4 and CS3 (the duration of the pubertal growth spurt), F – result of ANOVA, df – degrees of freedom

Type of malocclusion	CS4-CS3 [year]	F	df	*p* value
I N	1.07	0.947	1, 104	0.333*
III N	1.56			
I N	1.07	8.03	1, 107	0.006*
III H	2.68			
I N	1.07	4.61	1, 106	0.034*
I H	2.27			
III N	1.56	3.05	1, 99	0.084
III H	2.68			
III N	1.56	1.25	1, 98	0.265
I H	2.27			
III H	2.68	0.365	1, 101	0.547
I H	2.27			

## Discussion

Proper planning of orthodontic treatment is not possible without sound knowledge of craniofacial growth and development. The choice of a suitable method of treatment of the growing patient and treatment duration are fundamental for successful therapeutic outcome [27, 28]. Growth mechanisms pose a challenge since they depend on many processes. The potential and pattern of the craniofacial growth is determined genetically and environmentally [29]. Genetic mechanisms that modulate facial growth have not been fully recognized, and accurate gene mapping is still the question of future studies [30, 31]. Current studies on female twins indicate which structures rely more on genetic factors and which are more sensitive to environmental influences. Genes affect the shape of the nose, fullness of the lips, face size and pupillary distance, whereas environmental factors determine mandibular ramus height and horizontal facial asymmetry [32, 33]. The clinician, understanding the genetic and environmental background of the craniofacial growth and development, should also be aware of the proper duration of treatment of the young patient. Maxillary growth termination precedes the mandibular one; hence, all the therapeutic activities within the midface should be performed without delay [34, 35]. Mandibular growth, on the other hand, is related to facial muscle function and total rotation, which comprises the matrix and the intramatrix rotation. Additionally, one has to allow for compensation mechanisms [36]. Another critical factor is the variability of the rate of mandibular growth, with its accelerations and slowdowns [37]. From the perspective of dentofacial orthopaedics, the period of pubertal growth spurt, usually lasting several months, merits utmost attention due to the highest acceleration of mandibular growth in that period [27, 38].

There are a number of papers concerning the growth spurt period in patients with disturbed horizontal growth component. The majority of researchers confirm a longer period of pubertal growth spurt in patients with skeletal Class III [22, 23, 39]. The difference between patients with skeletal Class I and Class III is a matter of 4.8 to 5.9 months, which concurs with the results of the present study (6 months). The only discrepancy here is the absence of statistically significant differences between group III N and the others. The authors of previously published papers did reveal such differences. This discrepancy may be due to a different number of subjects in study groups, to standard deviation which has a higher value here (probably resulting from higher intersubject variability), and most of all a different method of statistical analysis. The linear model with interaction variables that has been adopted here seems the most suitable one regarding the cross-sectional character of the study and no uniformity of patients in stages CS3 and CS4.

Studies concerning the duration of pubertal growth spurt in high-angle patients are still scarce [25, 40]. There is one paper that compares skeletal Class I and II also in patients with skeletal open bite [41]. The number of high-angle patients is less numerous (83 subjects) in comparison with the present study (105). The results are similar to those presented here—they also confirm longer pubertal growth spurt in patients with skeletal open bite. The duration of pubertal growth spurt in normodivergent patients in the studies by Ghaleb et al. [41] is 1.109, which is almost identical with the results presented above (1.07). However, the difference between stage CS4 and CS3 for high-angle patients is smaller by 0.78 (approximately 10 months, if the I H group is taken into account). In both studies, the differences are statistically significant, and the discrepancy in the duration of growth spurt in patients with skeletal open bite may be related to a different climate zone. Turkey is subtropical with temperatures higher than those in Poland with its moderate climate. Puberty is affected by both environmental and genetic factors [42]. The Americans, for example, are shorter than the Dutch [1]. Some races or ethnic groups mature faster than others [43].

A study by Çelebi et al. [25] is another one that deals with pubertal growth spurt. The authors failed to find statistically significant differences between groups with different vertical and horizontal cephalometric parameters. However, they used the hand-wrist method acc. to Greulich and Pyle and not the CVM method, and the number of high-angle patients was lower (35 subjects). The fact that it was a longitudinal study works in its favour. The authors claim that cross-sectional studies cannot possibly reflect the actual changes in the human body, an observation that cannot be disputed. On the other hand, the purpose of the hand and wrist radiograph is only to determine the skeletal age. Therefore, prescribing it routinely for a young patient is contrary to radiological protection. A cephalometric radiograph will find its application in the diagnostics of malocclusions, treatment planning, monitoring its course, observation of growth and assessment of bone age [44]. It visualizes not only both jaws but also paranasal sinuses, the hard palate, tonsils and airways’ patency [45, 46]. Assessment of the cranial shape may be useful in diagnosing patients with genetic disorders [47]. Many ENT specialists, speech therapists, paediatricians and geneticists will find a cephalometric radiograph helpful. Being equipped with this multifunctional imaging tool, ordering an additional radiograph, merely to confirm the skeletal age, seems pointless. Many reports in literature confirm the repeatability of the CVM method [12, 13].

Differences in the duration of the pubertal growth spurt in different types of malocclusion are clinically significant. In open bites, an orthodontist will have more time at their disposal to undertake orthopaedic therapy consisting, for example, of intruding molars by means of occlusal pads or miniimplants. If this therapy is effective, it will prevent the patient from undergoing orthognathic surgery. It is our observation that in skeletal Class III, the increased growth potential will adversely intensify the skeletal defect. Awareness of the duration of the pubertal growth spurt will be useful when surgical procedures are planned. With the growth completed, the patient may hope for a more stable effect [48]. It has to be remembered that a teenager with a high-angle defect may grow longer than their normodivergent peer. In the present study, the oldest patients with skeletal open bite were almost 17.5 years old. These observations correlate with those by Behrents [26].

In females, and in the group comprising both sexes, the age for the CS4 stage statistically significantly differed in the following comparisons: I N vs. III N, I N vs. I H and I N vs. III H. A similar tendency was not observed in males, which may be due to fewer male participants in the study. Patients still tend to perceive malocclusions as an aesthetic defect; hence, girls’ parents are more interested in treatment of their offspring. The onset of growth spurt in males in comparison with females came half a year (I N) to 2 years later (III H), which is consistent with reports in literature [49].

The domination of females over males may have also affected the occurrence of the growth spurt earlier and minutely extend the duration of the growth spurt in both genders. The differences between the mean chronological age regarding CS4 and CS3 in males was lower than in females but the tendency in the duration of the growth spurt: normodivergent skeletal Class I < normodivergent skeletal Class III < high-angle skeletal Class I < high-angle skeletal Class III is recurring in females, males and in both genders.

Even though there were no publications involving bigger samples of high-angle patients when the present paper was being prepared, there is a clear need to examine larger populations from various clinical centres, or to perform meta-analysis covering a period of several years once new papers on this topic are published. In cross-sectional studies, the size of the sample is fundamental.

Several problems were encountered when the necessary material was being pooled. There were fewer number of cephalometric radiographs of patients with normal anteroposterior and vertical measurements than it had been expected (patients with skeletal Class I are infrequent attendees at the orthodontic clinics and they seldom have indications for having a cephalometric radiograph taken). Furthermore, patients with skeletal Class III were frequently excluded from studies since they had already commenced their orthodontic treatment, which is consistent with studies by Ghaleb et al. [41]. Moreover, high-angle patients were usually close to angle 38°, which means they were borderline and could not be included in the study due to NS/ML > 39° criterion.

## Conclusions

Based on the above results, pubertal growth spurt in high-angle patients persists statistically significantly longer than in normodivergent patients. The following tendency was observed in the duration of the pubertal growth spurt: normodivergent skeletal Class I < normodivergent skeletal Class III < high-angle skeletal Class I < high-angle skeletal Class III. This tendency has statistical significance only in high-angle patients in comparison with normodivergent skeletal Class I. The research also confirmed that pubertal growth spurt in males has its onset half a year to 2 years later than in females, depending on the defect.

## Supplementary information

ESM 1 (DOCX 31 kb)
